# Isolation and genetic characterization of *human coronavirus* NL63 in primary human renal proximal tubular epithelial cells obtained from a commercial supplier, and confirmation of its replication in two different types of human primary kidney cells

**DOI:** 10.1186/1743-422X-10-213

**Published:** 2013-06-27

**Authors:** John A Lednicky, Thomas B Waltzek, Elizabeth McGeehan, Julia C Loeb, Sara B Hamilton, Maya C Luetke

**Affiliations:** 1 Environmental and Global Health, College of Public Health and Health Professions, University of Florida, Box 100188, Gainesville, FL 32610-0188, USA; 2 Emerging Pathogens Institute, University of Florida, Gainesville, FL 32610, USA; 3 Infectious Diseases and Pathology, College of Veterinary Medicine, University of Florida, Bldg. 1379, Mowry Road, Gainesville, FL 32610, USA; 4 Stritch School of Medicine, Loyola University Chicago, 2160 S. First Ave, Maywood, IL 60153, USA; 5 Medical Countermeasures Division, MRIGlobal, 425 Volker Boulevard, Kansas City, MO 64110, USA

**Keywords:** RPTEC, SV40, CMV, HCoV-NL63, HHV-6B

## Abstract

**Background:**

Cryopreserved primary human renal proximal tubule epithelial cells (RPTEC) were obtained from a commercial supplier for studies of *Simian virus* 40 (SV40). Within twelve hrs after cell cultures were initiated, cytoplasmic vacuoles appeared in many of the RPTEC. The RPTEC henceforth deteriorated rapidly. Since SV40 induces the formation of cytoplasmic vacuoles, this batch of RPTEC was rejected for the SV40 study. Nevertheless, we sought the likely cause(s) of the deterioration of the RPTEC as part of our technology development efforts.

**Methods:**

Adventitious viruses in the RPTEC were isolated and/or detected and identified by isolation in various indicator cell lines, observation of cytopathology, an immunoflurorescence assay, electron microscopy, PCR, and sequencing.

**Results:**

Cytomegalovirus (CMV) was detected in some RPTEC by cytology, an immunofluorescence assay, and PCR. *Human Herpesvirus* 6B was detected by PCR of DNA extracted from the RPTEC, but was not isolated. *Human coronavirus* NL63 was isolated and identified by RT-PCR and sequencing, and its replication in a fresh batch of RPTEC and another type of primary human kidney cells was confirmed.

**Conclusions:**

At least 3 different adventitious viruses were present in the batch of contaminated RPTEC. Whereas we are unable to determine whether the original RPTEC were pre-infected prior to their separation from other kidney cells, or had gotten contaminated with HCoV-NL63 from an ill laboratory worker during their preparation for commercial sale, our findings are a reminder that human-derived biologicals should always be considered as potential sources of infectious agents. Importantly, HCoV-NL63 replicates to high titers in some primary human kidney cells.

## Background

Cell lines and primary cells obtained from commercial suppliers or through inter-laboratory transfer can contain adventitious (i.e., contaminating) viruses. This happens primarily because cytopathic effects (CPE) are not always apparent in virus-infected cell cultures, and consequently, the cells are unwittingly sold or transferred between laboratories [1]. The adventitious viruses that are encountered in cell cultures often stem from bovine serum that is used to supplement cell growth media, and include: bovine viral diarrhea virus (BVDV) [1-6], bovine polyomavirus [1,7,8], bovine parvovirus [1,9-11] (J. Lednicky, unpublished), and bovine herpes viruses [1,12-15]. Unintentional contamination of cultured cells by these serum-derived viruses has obvious consequences not only with regard to data generation, but also because it exerts a toll on time wasted in the performance of laboratory work, and the costs thereof. Other common sources of contaminating viruses are: (a) laboratory workers, and (b) animal-sourced enzymes (such as porcine trypsin) and (c) other biologicals that are used for cell culture [1]. Examples of viruses that stem from porcine trypsin that have recently been found as contaminants of many cell lines including those used for vaccine production are *Torque teno sus virus* (TTSuV), a member of the family *Anelloviridae,* and *Porcine circoviruses 1* and *2* (PCV1 and PCV2) [1,16-20]. Anelloviruses and circoviruses are relatively small viruses with single-stranded, circular DNA genomes that replicate within the nuclei of infected cells. CPE due to the presence of anelloviruses have not been well described at present. Finally, primary cells can contain endogenous retroviruses and other viruses. For example, primary monkey kidney cells, which are used for the detection of paramyxoviruses and picornaviruses in many American diagnostic microbiology laboratories, can contain endogenous simian viruses that are either latent in the kidneys, or cause persistent but inapparent kidney infections in their hosts [21].

The work described in this manuscript resulted from a previous study of SV40 transcription in primate cells (J. Lednicky, unpublished). SV40 is a polyomavirus that was once referred to as “vacuolating agent” or “Simian vacuolating virus 40” because commonly studied SV40 strains induce the formation of cytoplasmic vacuoles late during infection of most permissive primate cells [22]. A batch of primary human RPTEC that had been obtained for our previous transcription study of well-known vacuolating strains of SV40 proved unsuitable, as about 60% of the cells exhibited cytoplasmic vacuolation within 12 hours after they were seeded in flasks. Necrosis and apoptosis were also evident in some of the attached cells. Due to vacuolation and obvious cell deterioration, the RPTEC were rejected for our SV40 study. Nevertheless, as we often work with primary cells and continuously refine our research methodologies, we sought to determine a likely root cause(s) of the deterioration of the RPTEC to (a) Advance our understanding of primary cell culture technology, and (b) Explore whether proper biosafety practices were being observed. For example, might the RPTEC be contaminated with a significant pathogen best suited for work in biosafety level-3 or −4 laboratories?

We first tested whether vacuolation of the RPTEC stemmed from faulty media preparation. For example, vacuoles can form in Madin Darby Canine Kidney (MDCK) cells due to: (a) shortage of L-glutamine in the cell growth medium, (b) inappropriate addition of anti-fungal agents to the medium, (c) improper CO_2_ environment for the sodium bicarbonate concentration of the medium, (d) nutrient depletion of the medium, and (e) mycoplasma contamination [23]. Faulty media formulation was ruled out as the root cause of the failure of this batch of RPTEC to thrive. Instead, based on the progressive formation of CPE, the results of our initial diagnostic tests, and our cumulative experience with cell culture [1], we predicted that adventitious agents were causing the rapid demise of our RPTEC cultures. DNA extracted from the RPTEC tested negative by PCR for mycoplasma species, and polyomaviruses SV40 and BK virus (BKV), suggesting none of these was causing vacuolation and/or cell deterioration. However, a single cause of the RPTEC deterioration was unlikely, as we detected 3 different human viruses in the RPTEC: *Human cytomegalovirus* (CMV), *Human coronavirus* NL63 (HCoV-NL63)*,* and *Human herpesvirus* 6B (HHV-6B).

CMV, also known as *Human herpesvirus-5* (HHV-5), (subfamily *Betaherpesvirinae*), is a double-stranded DNA virus that establishes lifelong persistence; it can remain latent in different human tissues and is known to infect renal tubular epithelial cells. A majority of humans are seropositive for CMV [24,25]. Whereas CMV infections are typically asymptomatic in healthy humans, the virus can reactivate and cause disease in immunosuppressed patients, including those undergoing kidney transplantation. Indeed, CMV antigens and DNA are found in renal epithelial cells in kidneys of trauma victims examined during autopsy as well as in biopsies of renal allografts, indicating that these cells can harbor CMV in both healthy persons and allograft recipients [26,27]. HCoV-NL63 is a single-stranded positive-sense RNA virus of the genus *Alphacoronavirus* (family *Coronaviridae,* order *Nidovirales*). First identified in 2003 from a child with bronchiolitis in the Netherlands, it is now recognized that HCoV-NL63 can cause upper and lower respiratory tract infections in humans, primarily in infants and the elderly [28-33]. Wild-type HCoV-NL63 is difficult or impossible to isolate from clinical specimens in continuous cell lines [34], although the prototype HCoV-NL63 strain was propagated in LLC-MK2 cells [33] and in primary, differentiated human bronchial-tracheal respiratory epithelial cells cultured at the air-liquid interface [35]. There are at least three different HCoV-NL63 genotypes (A, B, and C) [34]. HHV-6B is a double stranded DNA virus (subfamily *Betaherpesvirinae*, genus *Roseolovirus*) that infects up to 100% of humans and is the causative agent of exanthem subitum, which is also known as roseola infantum or sixth disease [36]. After the primary infection, HHV-6B generally persists in latent form in T-lymphocytes and other cells. HHV-6B reactivation is common in transplant recipients, which can cause several clinical manifestations such as encephalitis, bone marrow suppression and pneumonitis [37].

The work presented herein serves as a reminder that biologicals (such as calf serum and cultured cells) can be contaminated with adventitious agents. The focus of this article is on the detection and isolation of HCoV-NL63, which to our knowledge, heretofore has not been reported in a natural infection of human kidney cells, or tested *in vitro* in primary human RPTEC.

## Results

### Initial observations

Within 12 hrs after cryopreserved RPTEC were thawed and seeded in cell culture flasks, we observed that about 60% of the attached cells were vacuolated. Since vacuolation may have been a sign of cytotoxicity due to residual cryopreservative, the RPTEC basal growth medium [basal growth medium (BGM)], which had been supplied with the cells, was changed. We noted by phase-contrast microscopy that prominent intranuclear inclusions surrounded by a clear halo (“owl-eyes”) were present in enlarged nuclei in some of the RPTEC, and that the same cells were enlarged relative to a majority of the others. These findings were considered pathognomonic for cytomegalovirus (CMV) [38] (Table 1).

**Table 1:** Indications of more than one virus in contaminated RPTEC

**Test performed**	**Cell line**^**f**^								
	**CV-1**	**HEK-293**	**LLC-MK2**	**MDCK**	**MDCK-London**	**Mv1 Lu**	**RPTEC**	**Vero E6**	**WI-38**
Microscopy, 12 hr post-seed	NA^a^	NA	NA	NA	NA	NA	Owl eye nuclei; enlarged cells	NA	NA
IFA, 48 hr post-seed	NA	NA	NA	NA	NA	NA	CMV positive	NA	NA
PCR, 48 hr post-seed	NA	NA	NA	NA	NA	NA	CMV positive^g^	NA	NA
BGM cytotoxicity	No effect	NT^c^	No effect	No effect	NT	NT	NA	No effect	No effect
Bioactive agent release assay	Vacuolation 12 hpi, 37°C	Vacuolation 12 hpi, 37°C	Vacuolation 12 hpi, 37°C	NT	NT	NT	NA	Vacuolation 12 hpi, 37°C	C.R., Sw. & Vac.^h^ 12 hpi, 37°C; cell death 48 hpi
Subcultures, 5 d post-seed of RPTEC	Vacuolation 12 hpi; CPE 6 dpi^b^	CPE 7 dpi^d^	Vacuolation 12 hpi; CPE 5 dpi^e^	CPE 6 dpi^e^	CPE 6 dpi^e^	CPE 6 dpi^e^	NA	Vacuolation 12 hpi; CPE 6 dpi^b^	C.R. Sw. & vac.^h^ 12 hpi, 37°C; cell death 48 hpi
Subcultures, freeze-thaw 7d post-seed of RPTEC	CPE 6 dpi^b^	CPE 7 dpi^d^	CPE 5 dpi^e^	CPE 6 dpi^e^	CPE 6 dpi^e^	CPE 5 dpi^e^	NA	CPE 6 dpi^e^	No CPE 30 dpi

^a^ NA, not applicable.

^b^ Vacuolation, formation of syncytia, rounding of the cells and detachment or cytolysis, at 34° and 37°C.

^c^ NT, not tested.

^d^ CPE consisting of rounding of the cells and detachment or cytolysis. Tested at 37°C [HEK-293 have reduced adherence at lower temperatures].

^e^ Vacuolation, formation of syncytia, focal rounding of the cells, formation of striations, or formation of syncytia followed by cytolysis, at 34° and 37°C., with or without TPCK-trypsin.

^f^ No CPE were detected in A549, NIH/3 T3, and BHK-21 cells for 1 month post-exposure to RPTEC-derived material.

^g^ PCR negative for HHV-1, HHV-2, BKV, and SV40.

^h^ C.R., Sw. & Vac., cell rounding, swelling and vacuolation.

**Figure 1:**
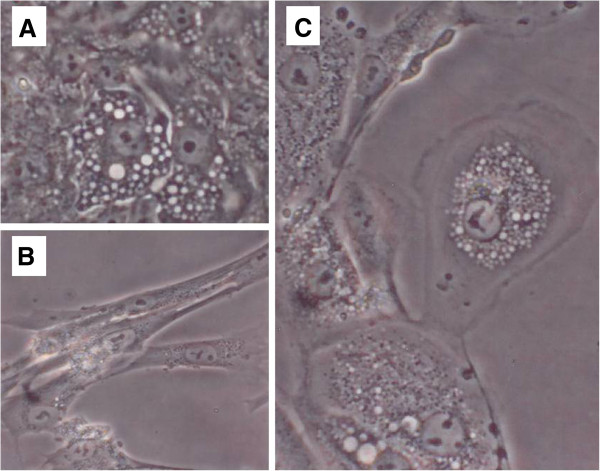
**Appearance of early RPTEC culture and of WI-38 cells during bioactive agent release assay.** [**A**] Vacuolated RPTEC cells, 24 hr culture, 400X. [**B**] Non-infected WI-38 cells demonstrating expected fibroblast shapes, 400X. [**C**] WI-38 cells 12 hrs post-exposure to spent BGM from a 24 hr RPTEC culture, 400X.

Vacuoles were still present 24 hrs post-seeding of the RPTEC (and after the RGM change at 12 hrs) (Figure 1A), but there were no signs of contamination by extracellular bacteria or fungi. The pH at 37°C of fresh BGM was approximately 7.36 (within normal range), and ammonia was not detected using a salicylate-based method (data not shown). These findings suggested neither incorrect pH nor presence of ammonia in BGM were causing vacuolation of the RPTEC.

Moreover, CV-1, LLC-MK2, and Vero cells, which are cell lines derived from monkey kidneys, did not get vacuolated after 24 hrs incubation with BGM. Thus, no evidence of cytotoxicity due to BGM was uncovered. By 36 hrs post-seed, vacuoles were still present in RPTEC in BGM that had been boosted with additional L-glutamine, suggesting glutamine deficiency was not an issue.

### Bioagent release assays

**Figure 2:**
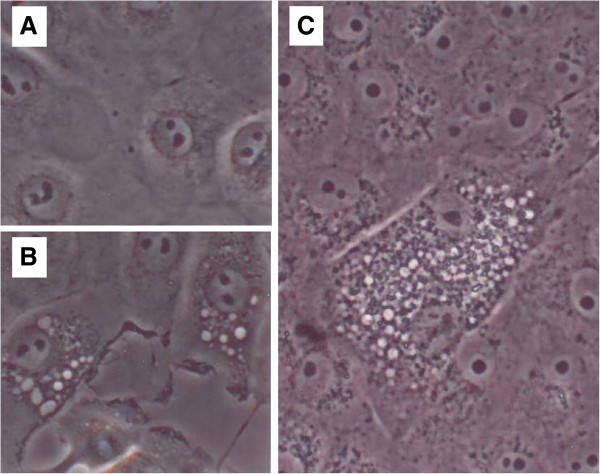
**Appearance of LLC-MK2 and Vero cells during bioactive agent release assay.** [**A**]. Normal LLC-MK2, 400X. [**B**] LLC-MK2 cells 12 hr after exposure to spent BGM from a 24 hr RPTEC culture, 400X. [**C**] Vero cells 12 hrs post-exposure to spent BGM from a 24 hr RPTEC culture, 400X.

A bioactive agent release assay indicated something in the spent BGM of the 24 hr RPTEC cultures induced enlargement and/or vacuolation of WI-38 (Figure 1B and C), LLC-MK2 (Figures 2A and B), Vero E6 cells (Figure 2C), and CV-1 and HEK-293 cells (not shown) within 12 hrs. Cell enlargement, rounding, and vacuolation were more notable in WI-38 cells than other cells (Table 1). These observations suggested the RPTEC were releasing either a biomolecule(s) or virus(es) that adversely affected some of the cell lines.

### Immunofluorescence assay (IFA) and PCR for CMV

Some RPTEC from 48 hr cultures were positive for CMV by IFA (their nuclei were fluorescent), and DNA extracted and purified from the cells also tested positive for CMV by PCR (data not shown). However, the extracted DNA was PCR negative for human herpes virus (HHV)-1 and HHV-2, and polyomaviruses SV40 and BKV (Table 1).

### Isolation of virus from live cells

**Figure 3:**
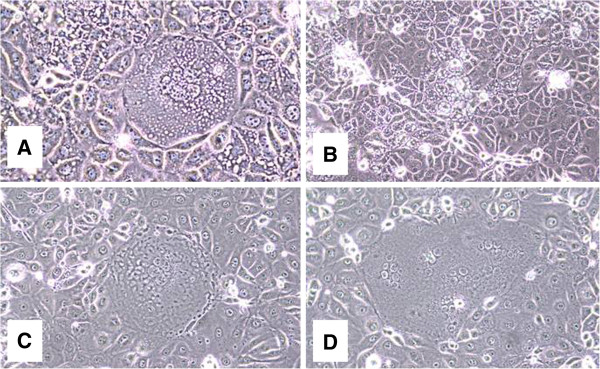
**Cytopathic effects in LLC-MK2 and MDCK cells inoculated with spent BGM from 5-day RPTEC cultures.** [**A**]. LLC-MK2 cells, 5 days p.i. 37°C (400X); vacuolated single cells and vacuolated syncytium are evident. [**B**] Foci of vacuolated LLC-MK2 cells, 4 days pi, 37°C (400X). [**C**,**D**] LLC-MK2 cells with large syncytia but few vacuoles, 5 days pi, 34°C (200X).

CPE consisting of cell swelling/rounding and/or vacuoles also occurred at 34° and 37°C in WI-38, CV-1, LLC-MK2, and Vero cells inoculated with spent media from 5-day old RPTEC cultures. As for the bioagent release assay, morphological aberrations were most notable in WI-38 cells. Trypsin did not enhance CPE in LLC-MK2, MDCK, MDCK-London, Mv1 Lu, or Vero cells. The WI-38 cells (but not the other cells) died 2 days afterwards. However, starting day 5 post-inoculation (p.i.), occasional syncytia with 8 or more nuclei were noted in LLC-MK2, CV-1, HEK-293, Mv1 Lu, and Vero cells, and smaller syncytia (with up to 8 nuclei) in MDCK and MDCK-London cells (Table 1). Thereafter, CPE were most pronounced in LLC-MK2 cells and in HEK293 cells. In LLC-MK2 cells, most of the early CPE consisted of vacuolation and the formation of foci of detached rounded cells, many forming elongated oblong clumps of rounded cells above the monolayer (referred to as “striations” in ref. [50]). At later times, cytolysis of syncytia occurred. Vacuolation in LLC-MK2 cells appeared more pronounced at 37° than 34°C, and conversely, syncytia appeared larger at 34° than 37°C (Figures 3A-D). Rounding followed by eventual detachment from the growing surface occurred in infected HEK-293 cells (not shown). In MDCK cells, vacuoles were also more pronounced at 37° than 34°C.

### Electron microscopy of contaminated RPTEC

**Figure 4:**
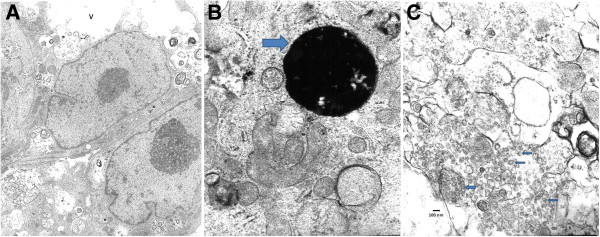
**Transmission electron micrographs of contaminated RPTEC cells.** [**A**] Contaminated RPTEC cells, 5 day culture, original magnification 6000x. Vacuoles (v) and evidence of cell deterioration are evident. [**B**] Round, electron dense cytoplasmic inclusion (blue arrow) in 5 day old culture of contaminated RPTEC cells, original magnification35,000x. [**C**] Free virus-like particles (thin arrows), and virus-like particles in a vesicle (thick arrow) from a 5 day culture of contaminated RPTEC cells, original magnification 35,000x.

Due to cell vacuolation and deterioration, electron micrographs of five day old RPTEC cultures were difficult to interpret. At low magnification, vacuoles and cell deterioration were obvious (Figure 4A). Occasional viral particles consistent in appearance and size with CMV at different stages of maturation were observed at higher magnifications (data not shown). In addition to nuclear inclusions, homogenous electron-opaque, dense cytoplasmic bodies were present. However, unlike the irregular-shaped cytoplasmic bodies we usually observe in CMV-infected cell cultures (J. Lednicky, unpublished observations), these were distinctly circular, as described by Smith and de Harven for CMV in infected cells [39] (Figure 4B). Additionally, we also noted many virus-like particles (VLP) that were morphologically different from CMV; these VLP were present as free particles, within vacuoles, and in transport vesicles. A majority of the virus-like particles were spherical, and they collectively ranged from about 80 to 120 nm in diameter, and some seemed to have surface projections (Figure 4C).

### Virus isolation from freeze-thawed RPTEC

Somewhat different results were obtained when the indicator cells of Table 1 were inoculated with freeze-thawed RPTEC lysate from 7-day old cultures instead of spent media from 5-day old RPTEC cultures. In contrast to previous findings, CPE were not observed in WI-38 cells at early times onto 30 days p.i. However, CPE were seen in LLC-MK2 cells starting 4 days p.i., and in other cells at later times (Table 1). As before, vacuolation was more pronounced at 37° than 34°C.

Since syncytia were observed, we focused PCR efforts on the detection of the viruses that we considered the most likely candidates: coronaviruses, human paramyxoviruses, and reoviruses (HHV-1 and −2 were already ruled out, section 3, above). We did not test for retroviruses, acknowledging that exogenous or endogenous retroviruses may have been causing syncytia in the cells. Extracted nucleic acids were tested by PCR or RT-PCR using assays designed to detect known human coronaviruses [33,40-42], paramyxoviruses [43-45], and reoviruses [46].

**Figure 5:**
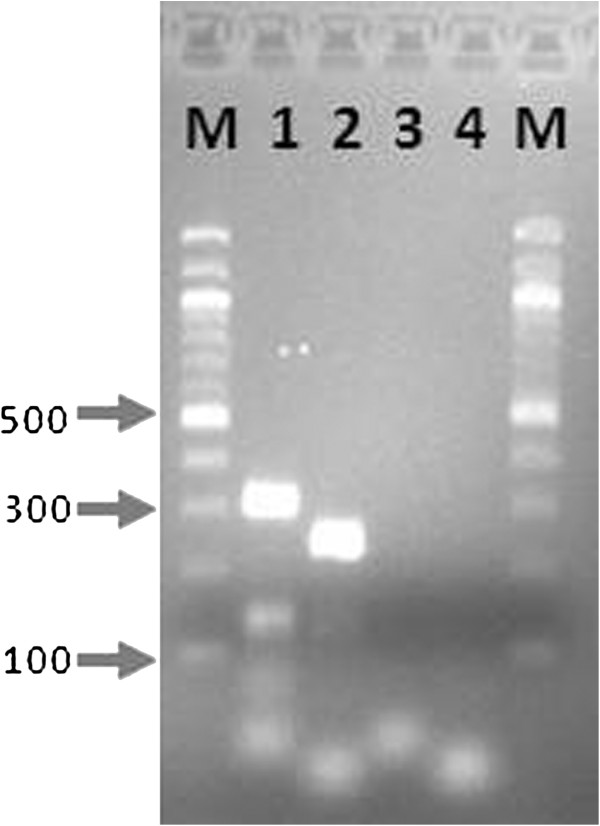
**RT-PCR detection of HCoV-NL63 in LLC-MK2 cells.** Lane M, 100 bp MW markers (New England Biolabs); Lane 1, HCoV-NL63-specific PCR product (314 bp) amplified by PCR primers N5-PCR1 and N3-PCR1 [33]; Lane 2, HCoV-NL63-specific PCR product (237 bp) amplified by PCR primers repSZ-1 and SZ-3[33]; Lane 3, Non-infected LLC-MK2 control tested using PCR primers N5-PCR1 and N3-PCR1; Lane 4, Non-infected LLC-MK2 control tested using PCR primers repSZ-1 and SZ-3.

RT-PCR and sequencing showed one of the viruses in the CV-1, HEK-293, LLC-MK2, MDCK, MDCK-London, Mv1 Lu, and Vero E6 cells was coronavirus HCoV-NL63. An example of RT-PCR reactions performed with 2 primer sets specific for HCoV-NL63 is shown in Figure 5.

### Electron microscopy of HCoV-NL63 in LLC-MK2 cells

**Figure 6:**
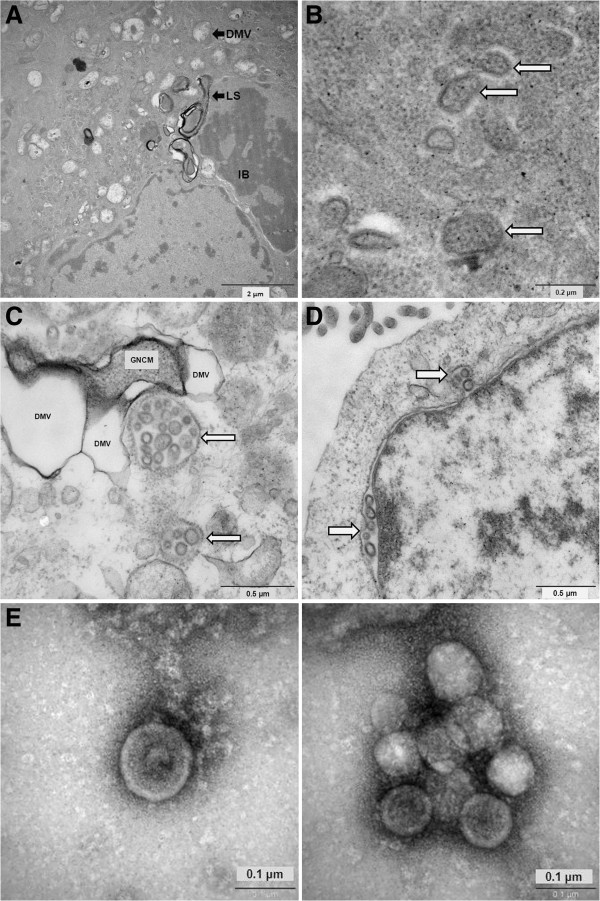
**Transmission electron micrographs of HCoV-NL63 in LLC-MK2 cells.** Scale bars are shown at the bottom right of each figure. [**A**] Intracellular structures typical of those formed in HCoV-NL63 infected cells: double membrane vacuole (DMV), laminar structure (LS), and inclusion body (IB). Original magnification 10,000x. [**B**] Granular nucleocapsid material in packets (arrows) typical of those formed by HCoV-NL63 in infected cells. Original magnification 100,000x. [**C**] Immature HCoV-NL63 particles in rough endoplasmic reticulum (RER) cisternae, with ribosomes in place (large arrows). Electron dense granular nucleocapsid material is visible in some of the virus particles. Double membrane vacuoles (DMV) are evident, adjoining a granular nucleocapsid material in a packet (GNCM), in association with the larger packet of virus particles. Original magnification 50,000x. [**D**] Immature HCoV-NL63 particles in RER adjacent to the nucleus of an infected cell. Original magnification 40,000x. [**E**] Free (mature) HCoV-NL63 particles (80 – 100 nm) in spent media. Original magnifications at 200,000x.

Proof that HCoV-NL63 was replicating in the LLC-MK2 cells was obtained by electron microscopy (Figure 6A-E). Characteristic features of HCoV-NL63 replication in LLC-MK2 cells [35,47] were detected, such as the formation of double membrane and laminar structures, and inclusion bodies (Figure 6A). Packets of granular nucleocapsid material were also evident in infected cells (6B). Virus particles at various stages of maturation were present in the cytoplasm (6C) and in the RER outside the nuclei (6D). Free virus particles 80 – 100 nm in diameter were present in spent media (6E). A counter-stain was not used to easily visualize the viral spikes (“crown”) surrounding the viruses in Figure 6E.

### Molecular dataset, sequence alignment, and phylogenetic analysis

**Figure 7:**
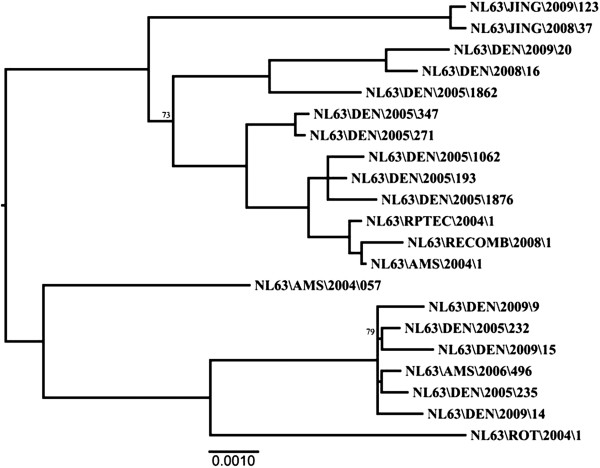
**Phylogram depicting the relationship of NL63 coronavirus isolate RPTEC/2004/1 to representative NL63 isolates.** Bayesian tree based on the full length genomic sequences (27,490 characters including gaps) for 21 NL63 coronavirus isolates. All nodes were supported by a posterior probability of > 95 unless otherwise noted. Branch lengths are based on the number of inferred substitutions, as indicated by the scale. Genomic sequences were obtained from GenBank: NL63/JING/2009/123 (accession number JX524171), NL63/JING/2008/37 (JX104161), NL63/DEN/2009/20 (JQ765567), NL63/DEN/2008/16 (JQ65566), NL63/DEN/2005/1862 (JQ765574), NL63/DEN/2005/347 (JQ765572), NL63/DEN/2005/271 (JQ765571), NL63/RECOMB/2008/1 (FJ211861), NL63/AMS/2004/1 (AY567487), NL63/DEN/2005/1062 (JQ765573), NL63/DEN/2005/193 (JQ765568), NL63/DEN/2005/1876 (JQ765575), NL63/AMS/2004/057 (DQ445911), NL63/DEN/2009/9 (JQ765563), NL63/DEN/2009/14 (JQ765564), NL63/DEN/2009/15 (JQ765565), NL63/DEN/2005/232 (JQ765569), NL63/DEN/2005/235 (JQ765570), NL63/AMS/2006/496 (DQ445912), NL63/ROT/2004/1 (AY518894), NL63/RPTEC/2004/1 (JX504050).

The complete consensus genomic sequence of HCoV-NL63 was obtained for virus in LLC-MK2 cells that had been incubated at 34°C. The virus, designated HCoV-NL63 strain RPTEC/2004/1, has a genomic length of 27,553 bp, and the complete sequence has been deposited in GenBank (accession no. JX504050). A dataset was prepared containing complete or nearly complete HCoV-NL63 genomes in GenBank. To construct a phylogram, the final aligned genomic dataset contained 27,490 nucleic acid characters (including gaps) for 21 unique HCoV-NL63 isolates. A jModeltest identified the GTR+I+G model to be the most suitable model for phylogenetic analyses. The results of the genomic phylogenetic analysis revealed the newly sequenced coronavirus isolate NL63/RPTEC/2004/1 is most closely related to a 2004 Amsterdam isolate, and some American isolates from 2005 (Figure 7).

### PCR detection of another herpesvirus in DNA from contaminated RPTEC

For more comprehensive analyses. PCR tests for herpesviruses that were not included in our previous assays (for HHV-3,-4,-6,-7, and −8) were performed on DNA extracted from RPTEC. A 151-bp amplicon was generated using nested primers for HHV-6 [48]. Identity was confirmed by sequencing (data not shown).

### Biotypes of plaque-purified HCoV-NL63/RPTEC/2004 compared to HCoV-NL63/Amsterdam-1

**Figure 8:**
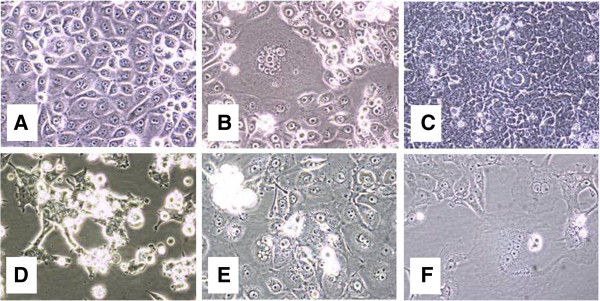
**Cytopathic effects formed by HCoV-NL63/RPTEC/2004 pp A and by HCoV-NL63 Amsterdam −1.** [**A**]. Non-infected LLC-MK2 cells, 8 days, 33°C (400 X). [**B**] LLC-MK2 cells infected with HCoV-NL63/RPTEC/2004 pp A, 8 days p.i., 33°C, showing a syncytium, rounding of some cells, and areas of clearing (400X). [**C**] Non-infected HEK-293 cells, 8 days, 37°C (400X). [**D**] Advanced cytopathic effects in HEK-293 cells 8 days p.i. with HCoV-NL63/RPTEC/2004 ppA, 37°C (400X). [**E**] LLC-MK2 cells infected with HCoV-NL63/Amsterdam-1, 33°C, 6 days p.i; detached cells, areas of clearing, vacuolation, and a small syncytium are visible (400X). [**F**] Vero cells infected with HCoV-NL63/Amsterdam-1, 33°C, 8 days p.i.. Vacuolation, a few floating dead cells, large areas of clearing, and a small syncytium are visible (400X).

**Figure 9:**
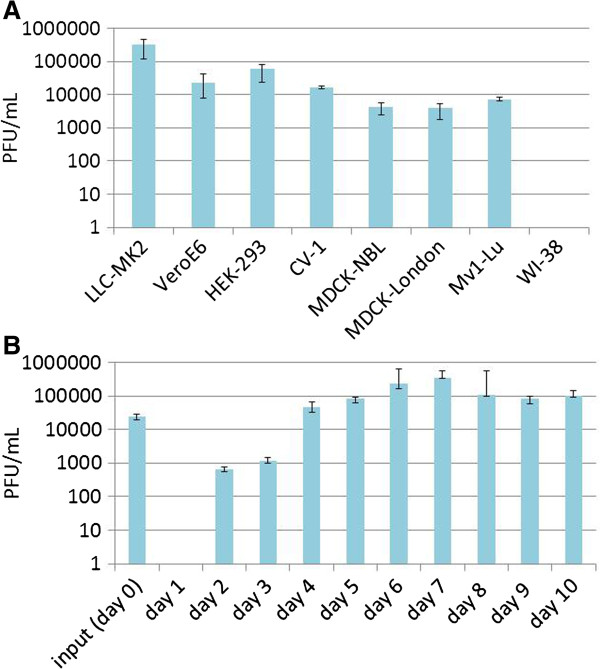
**HCoV-NL63/RPTEC/2004 titers in cultured cells.** [**A**]. Virus titers seven days post-infection of indicator cells infected with HCoV-NL63/RPTEC/2004 pp A. Titers were obtained from free virus in spent media; plaque assays were performed in CaCo-2 cells. Average virus titers (PFU/ml, mean of 3 measurements) were: LLC-MK2 cells, 3.2 × 10^5^; Vero E6 cells, 2.3 × 10^4^; HEK-293 cells, 5.9 × 10^4^; CV-1 cells, 1.6 × 10^4^; MDCK-NBL cells, 4.3 × 10^3^; MDCK-London cells, 4.1 × 10^3^; Mv1 Lu cells, 6.9 × 10^3^; WI-38 cells, none detected. [**B]**. Virus production over nine days by NL63/RPTEC/2004 pp D in LLC-MK2 cells. Titers (PFU/ml) peaked on day 6, and remained in the low 10^5^ range thereafter until day 9 p.i. (last day of measurement).

Since it was likely that multiple viruses contributed to the observations described in Table 1, an attempt was made to plaque purify HCoV-NL63 in CaCo-2 cells [49] at 37°C [29,49] and in LLC-MK2 cells at 32°C (32° to 34°C are considered optimal temperatures for the *in-vitro* cultivation of HCoV-NL63 [33-35,47,50,51]). Whereas HCoV-NL63 replicates more effectively in CaCo-2 cells than LLC-MK2 cells [49], that information was not available and therefore CaCo-2 cells were not used in our initial studies (Table 1), which were performed in 2004. Nine days p.i., LLC-MK2 cells were stained with neutral red, individual plaques picked, and subjected to 1 more round of plaque purification [50]. Similarly, foci of CPE were identified under an unstained agarose overlay in CaCo-2 cells 5 days p.i., picked, and subjected to 2 more rounds of plaque purification [49]. Plaque-purified stocks resulting from LLC-MK2 (NL63/RPTEC/2004 pp A – C) or CaCo-2 (NL63/RPTEC/2004 pp D – F) were chosen for biotype analyses after confirming they were PCR negative for CMV and HHV-6B. After titration of the plaque-purified HCoV-NL63/RPTEC/2004 stocks in LLC-MK2 cells, the cells of Table 1 were infected at a MOI of 0.1 PFU/cell. HCoV-NL63/RPTEC/2004 pp A – F) formed the same CPE described in Table 1 that were observed for freeze-thawed RPTEC, though formation of CPE was delayed by at least 1 day. A few examples are depicted in Figure 8A-C. Similarly, HCoV-NL63/Amsterdam-1 that had been plaque purified in LLC-MK2 cells formed the same type of CPE as the plaque-purified HCoV-NL63/RPTEC/2004 isolates (Figure 8D-F). In brief, each plaque-purified virus induced vacuolation, rounding of the cells, and the formation of syncytia in LLC-MK2 and Vero cells. Striations occurred at early times post-infection in LLC-MK2 cells, and to a lesser extent in HEK-293 cells. CPE were least obvious in MDCK and Mv1 Lu cells. With the exception of HEK-293 cells, which were only tested at 37°C (below 35°C, these cells do not adhere well to the growing surface of a flask), CPE were first detected at 33°C. From spent media harvested from 7-day old cultures, viral titers were obtained for HCoV-NL63/RPTEC/2004 pp isolates A – F and HCoV-NL63-Amsterdam-1 using plaque assays in CaCo-2 cells [49]. For each cell line that was tested (as listed in Table 1), the viral titer was similar for each virus. Representative results, obtained for HCoV-NL63/RPTEC/2004 pp isolate A (Figure 9A), indicate the highest titer (3.2 × 10^5^ PFU/mL) was attained when the virus was propagated in LLC-MK2 cells. Using a MOI of 0.1 PFU/cell, we tested progeny virus production by HCoV-NL63/RPTEC/2004 pp A and D in LLC-MK2 cells. The virus yields over a 9-day infection period were determined by plaque assays in CaCo-2 cells. Similar results were obtained for the 2 viruses; the results for RPTEC/2004 pp D are shown in Figure 9B.

### Growth of HCoV-NL63/RPTEC/2004- and -Amsterdam-1 in primary human kidney cells

**Figure 10:**
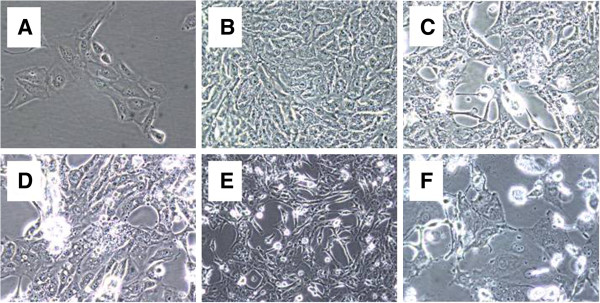
**Cytopathic effects in a new batch of primary RPTEC infected with HCoV-NL63/RPTEC/2004 pp A and HCoV-NL63/Amsterdam-1.** [**A**] Subconfluent RPTEC, 400x. [**B**] Non-infected confluent RPTEC, 400x. [**C**] Confluent RPTEC infected with HCoV-NL63/RPTEC/2004 pp A, 3 dpi, 400x. [**D**] Confluent RPTEC infected with HCoV-NL63/Amsterdam-1, 3 dpi, 400x. [**E**] Confluent RPTEC infected with HCoV-NL63/RPTEC/2004 pp A, 3 dpi, 200x. [**F**] Confluent LLC-MK2 cells infected with HCoV-NL63/RPTEC/2004 pp A from new RPTEC, 3 dpi, 400x.

**Figure 11:**
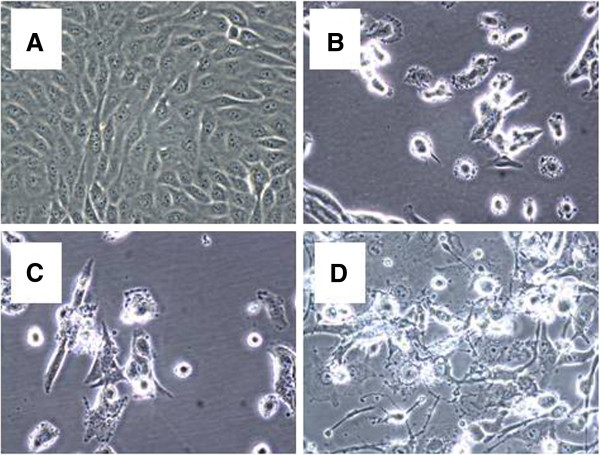
**Cytopathic effects in primary HRE cells infected with HCoV-NL63/RPTEC/2004 pp A or HCoV-NL63/Amsterdam-1.** [**A**] Non-infected confluent HRE, 400x. [**B**] Confluent HRE infected with HCoV-NL63/RPTEC/2004 pp A, 3 dpi, 400x. [**C**] Confluent RPTEC infected with HCoV-NL63/Amsterdam-1, 3 dpi, 400x. [**D**] Confluent LLC-MK2 cells infected with HCoV-NL63/RPTEC/2004 pp A from new RPTEC, 3 dpi, 400x.

Newly acquired (in 2013) primary RPTEC, HRE, and HRCE cells did not release a detectable bioagent (data not shown). What may have been “owl’s eye” nuclei were observed rarely only in HRE cells. Both HCoV-NL63/RPTEC/2004 and HCoV-NL63/Amsterdam-1 caused rapid formation of CPE in RPTEC (Figure 10) and HRE cells (Figure 11) infected at a MOI of 0.1 with plaque purified HCoV-NL63/RPTEC/2004 pp A or HCoV-NL63/Amsterdam-1. We noted that the RPTEC were not vacuolated when sub-confluent (Figure 10A) yet became vacuolated once confluent (Figure 10B), but otherwise stayed viable when re-fed every 2 days with REBM. Extensive CPE consisting of rounding of the cells and cytolysis occurred by 3 dpi in RPTEC (Figure 10C-E) and 4 dpi in HRE cells (Figure 11B-C). When 1 ml of spent REBM was obtained from RPTEC or HRE cells 3 days after they had been infected with HCoV-NL63 RPTEC/2004 pp A or HCoV-NL63/Amsterdam-1, and inoculated onto LLC-MK2 cells in T25 flasks, CPE were extensive 3 days later (Figure 10F and Figure 11D). In contrast, 1 ml of spent media from non-infected (negative control) RPTEC and HRE cells had no effect on LLC-MK2 cells (data not shown). The presence of HCoV-NL63 in the spent media of RPTEC and HRE that had been inoculated with the viruses, and in the indicator LLC-MK2 that had been inoculated with spent media from the virus-infected cells, was confirmed by RT-PCR (data not shown). In contrast, CPE were sparse in HRCE cells 7 dpi with either HCoV-NL63/RPTEC/2004 pp A or HCoV-NL63/Amsterdam-1 (data not shown).

### Virus titers in primary human cells

**Figure 12:**
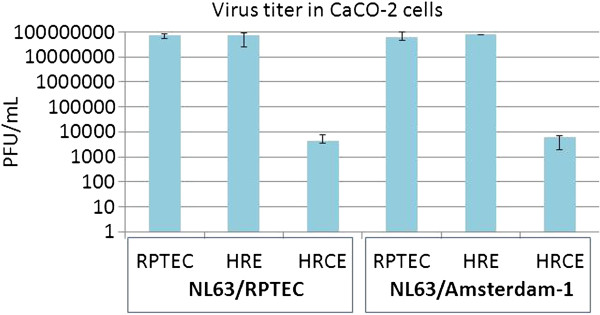
**Virus titers three days post-infection of primary cells infected with HCoV-NL63/RPTEC/2004 pp A or HCoV-NL63/Amsterdam-1.** Titers were obtained from free virus in spent media; plaque assays were performed in CaCo-2 cells. Average virus titers for HCoV-NL63/RPTEC/2004 pp A (PFU/ml, mean of 3 measurements) were: RPTEC, 6.9 × 10^7^; HRE cells, 6.4 × 10^7^; HRCE cells, 5.2 × 10^3^. Average virus titers for HCoV-NL63/Amsterdam-1 (PFU/ml, mean of 3 measurements) were: RPTEC, 6.9 × 10^7^; HRE cells, 7.8 × 10^7^; HRCE cells, 5.1 × 10^3^.

Both HCoV-NL63/RPTEC/2004 and HCoV-NL63/Amsterdam-1 formed relatively high viral titers by 4 dpi in RPTEC and HRE cells, but not in HRCE cells (Figure 12). The viral titers exceeded those formed in LLC-MK2 cells by about 2 orders of magnitudes (ie, by 2 logs). In contrast, viral titers for both virus strains remained low (10^3^ PFU/ml) in HRCE cells by 9 dpi (data not shown).

## Discussion

The presence of CMV in the original batch of virus-contaminated RPTEC was not a surprise to us, as we have isolated CMV from frozen (−80°C) simian kidneys and from primary simian kidney cells (Lednicky, unpublished). We learned from the supplier that the donor of the virus-contaminated RPTEC of this study was seropositive for CMV. However, our batch of virus-contaminated RPTEC was not checked for the presence of CMV by the supplier (personal communication). As precedence for the presence of CMV in human kidney cells *in vivo*, it is known that reactivation of CMV in renal tubule epithelial cells can complicate kidney transplantation, leading to poor long-term graft function [52]. The apparent complete inactivation of CMV by the freeze-thaw procedure we used was unexpected, as the process does not always completely inactivate CMV [53], but was nevertheless fortuitous, leading to observations resulting in the detection of HCoV-NL63. Then again, it may have inactivated other viruses in the RPTEC.

To our knowledge, ours is the first description of HCoV-NL63 in primary RPTEC. Overall, our observations of HCoV-NL63 growth in various cell lines appear consistent with literature reports. Growth of the virus in LLC-MK2 and Vero cells is well known [29,33,54]. The ability of the virus to form CPE in MDCK was previously described [54]. The lack of HCoV-NL63 growth in human fibroblasts has been reported [54]. In particular, MRC-5 cells, did not support the replication of HCoV-NL63 [54], and those cells are used interchangeably with WI-38 cells in American diagnostic virology laboratories for the isolation of respiratory viruses and CMV (both cell lines are derived from human fetal lung cells). Thus, it is not surprising that HCoV-NL63 does not replicate in WI-38 cells. Growth of HCoV-NL63 at 37°C has been reported and should not be a surprise [29,49]. That HCoV-NL63 might induce vacuolation is not a surprise, as that is a common property of coronaviruses. It will be interesting to see if interaction with ganglioside GM1 is related to the vacuolation process, as reported for SV40 [22].

HCoV-NL63 replicated in HEK-293 cells, as does SARS-CoV [55,56]. Both SARS-CoV and HCoV-NL63 can use angiotensin-converting enzyme 2 *(*ACE2*)* as a viral receptor [57], and ACE2 is expressed in kidneys [58], and may be reasons HCoV-NL63 was present in our batch of RPTEC and could infect HEK-293 cells. Replication of SARS-CoV in Mv1 Lu cells was previously reported [59], so perhaps it is not surprising that HCoV-NL63 does as well, if the viruses share receptor specificity, and Mv1 Lu cells contain the cellular machinery necessary for the replication of these viruses. However, the origin of HEK-293 is unclear, as the cells express neurofilament (NF) subunits NF-H, NF-L, NF-M, alpha-internexin, and other proteins found in neurons [60]. Thus, HEK-293 may be of neuronal origin, and it will be interesting in the future to discern which neural and kidney cells support the replication of HCoV-NL63.

It is not clear why rapid cell swelling rounding, and vacuolation, followed by cell death, occurred in WI-38 cells. Our current hypothesis is that CMV was latent in the kidney cells of the donor of the RPTEC, and that the virus was reactivated during the initial harvest of cells from the donor’s kidney. We surmise that within our batch of RPTEC, that many of the cells had been inadvertently frozen when they were at an early stage of CMV infection. It is likely that the cells produced a large yield of CMV when they were brought out of cryopreservation, and that the high-titer CMV infected the permissive WI-38 at a high MOI, and this resulted in rapid killing of those cells. Since we were unprepared for such analyses, a quantitative enumeration of infectious CMV particles was not performed. We also suspect that CMV from the RPTEC had infected Vero, LLC-MK2, and CV-1 cells, but the infection was abortive [38], unlike the situation in WI-38 cells, which are permissive for that virus.

Finding that the HCoV-NL63 is similar to viruses from 2004 and 2005 is perhaps not surprising, as the RPTEC of this report were prepared from a donor and purchased (by us) that same year.

To our knowledge, HCoV-NL63 has not been reported in *natural* infections of human kidneys. The ability of HCoV-NL63 to replicate to high titers in primary RPTEC and HRE cells suggests that at least some human kidney cells are fully permissive for the virus. However, we are unable to resolve whether (a) The original batch of contaminated RPTEC were infected (naturally) with the virus *prior* to harvest, or (b) A worker with a respiratory infection accidentally contaminated the RPTEC during their initial preparation, or (c) The RPTEC were contaminated in our laboratory. We are unable to resolve the issue whether the cells were contaminated during preparation for many reasons, foremost being the company that sold the cells was merged with a different entity. It is unlikely that the RPTEC were infected in our laboratory, as we did not have HCoV-NL63 in our laboratory in 2004, and acquired HCoV-NL63/Amsterdam-1 only recently (Sept. 2012) so that we could compare the biotype of HCoV-NL63/RPTEC with that of Amsterdam-1. Moreover, our laboratory policy dictates that workers refrain from cell culture work when they have a respiratory tract infection. It is plausible (but we lack proof) that HCoV-NL63 may have been latent in the donor’s kidneys, a possibility consistent with the known biology of various coronaviruses that establish long-term but sub-clinical infections. Noteworthy, SARS-CoV, which shares the same ACE2 receptor as HCoV-NL63, has been associated with kidney disease [61-64]. SARS-CoV causes a systemic infection with viral shedding not only in respiratory secretions, but also in stool and urine [63,65,66]. Perhaps HCoV-NL63 is capable of causing systemic infections as well, though the severity is much less than that of SARS-CoV. A parallel to this notion is the finding that HCoV-NL63 replicates to high titers in CaCo-2 cells [49], which are derived from a human colon carcinoma. In April of 2012, a new coronavirus capable of causing severe acute respiratory infections of humans emerged in Jordan. The same coronavirus was isolated in the summer of 2012 from a patient with acute pneumonia and renal failure in Saudi Arabia [67,68]. The new virus has been fully sequenced, classified as a group C β-coronavirus [69-71], and termed Middle East Respiratory Syndrome Coronavirus (MERS-CoV) by the Coronavirus Study Group of the International Committee on Taxonomy of Viruses (announced in J. Virology on May 15, 2013). Genetically, MERS-CoV is closely related to SARS-CoV, and is another example of a coronavirus associated with respiratory disease that can also infect kidney cells. The donor of the RPTEC of our study did not have kidney disease (otherwise, the cells would not have been harvested and sold for research purposes), suggesting a persistent, sub-clinical infection of the kidneys by HCoV-NL63 is more likely.

To what extent, if any, HHV-6B may have somehow modulated the growth of the other viruses in the RPTEC is unclear. Noteworthy, HHV-6B has also been reported in association with renal epithelial cells and kidney transplant rejection [72].

Lastly, whereas the virus-like particles of Figure 4C appear similar to those in an electron micrograph of SARS-CoV in kidney tissue [63], we have no formal proof that they are in fact HCoV-NL63 and may be another virus we did not identify in our work. Taken together, our findings are a reminder that human-derived biologicals should always be considered as potential sources of infectious agents. Moreover, our findings raise the possibility of kidney involvement during the course of infection with HCoV-NL63.

## Materials and methods

### Cells and cell-growth media

Cryopreserved primary human RPTEC were obtained from a commercial source in the USA. BGM, supplements, and growth factors [fetal bovine serum, insulin, transferrin, triiodothyonine (T3), human recombinant epidermal growth factor, hydrocortisone, epinephrine, gentamicin sulfate, and amphotericin-B] were concurrently obtained as a kit from the RPTEC supplier. The RPTEC were first seeded onto four T25 flasks and manipulated following instructions included with the kit. MDCK-London cells were a gift from Dr. Gary Heil, University of Florida. Cell lines A549 (CCL-185), BHK-21 (CCL-10), CaCo-2 (HTB-37), CV-1 (CCL-70), HEK-293 (CRL-1573), LLC-MK2 (CCL-7), MDCK, (CCL-34), Mv1 Lu (CCL-64), NIH/3 T3 (CRL-1658), Vero E6 (CRL-1586), and WI-38 (CCL-75) were obtained from the ATCC (Manassas, VA), and along with MDCK-London cells, were propagated as monolayers at 37°C and 5% CO_2_ in Dulbecco's Modified Eagle's Medium (DMEM) (Mediatech, Inc., Manassas, VA) or Eagle’s Minimal Essential Medium (EMEM) (Invitrogen Corp., Carlsbad, CA), as appropriate per cell line. DMEM and EMEM were initially supplemented with 2 mM L-Glutamine, which was later substituted with 2 mM L-Alanyl-L-Glutamine (GlutaMAX™, Invitrogen Corp.). Both DMEM and EMEM were supplemented with antibiotics [PSN; 50 μg/ml penicillin, 50 μg/ml streptomycin, 100 μg/ml neomycin (Invitrogen Corp.)], and 10% (v/v) low IgG, heat-inactivated gamma-irradiated fetal bovine serum (HyClone, Logan, UT). Additionally, sodium pyruvate (Invitrogen Corp.) and non-essential amino acids (Hyclone) were added to EMEM., with the exception: EMEM formulated with calf serum (HyClone) instead of FBS was used for NIH/3 T3 cells. Before seed stocks were prepared, the cell lines were propagated in growth media with plasmocin (Invivogen, San Diego, CA) for 2 weeks to reduce the chances of mycoplasma contamination. Next, the cell lines were incubated for a minimum of 2 weeks in the absence of antibiotics to determine whether fast-growing microbial contaminants were present or abnormal morphological changes would occur (associated with intracellular mycoplasma). Following 2–3 weeks of propagation without antibiotics, the plasmocin-treated cell lines and RPTEC cells were tested by PCR for the presence of mycoplasma DNA using a Takara PCR Mycoplasma Detection kit (Fisher Scientific, Pittsburgh, PA) [1]. The cells tested negative for mycoplasma. An independent laboratory (at the University of Florida) confirmed that the stock of LLC-MK2 cells that was used for the isolation of HCoV-NL63 in this manuscript was negative for human respiratory viruses including human coronaviruses 229E, HKU1, OC43, and NL63 using a GenMark multiplex respiratory PCR eSensor XT-8 Respiratory Viral Panel (eSensor RVP; GenMark Diagnostics, Inc., Carlsbad, CA).

### Glutamine deficiency test

Fresh L-glutamine was added to BGM in a 24 hr RPTEC culture and the cells observed every six hrs for one day to assess the effect on cell morphology, vacuolation, and viability.

### BGM cytotoxicity assay

Complete, freshly prepared BGM was substituted for DMEM in subconfluent cultures of CV-1, LLC-MK2, MDCK, Vero, and WI-38 cells, and the cells incubated at 37°C and observed every 12 hours over 3 days for morphological changes or cell death as evidence of cytotoxicity.

### Bioactive agent release assay

To find out whether the RPTEC were releasing a bioactive agent, spent BGM from a 24 hr RPTEC culture was equally subdivided and added to subconfluent CV-1, HEK-293, LLC-MK2, Vero E6, and WI-38 cells in T-25 flasks. These particular cell lines were chosen on the assumption that a virus growing in RPTEC would preferentially infect primate over non-primate cells. After inoculation, the cells were incubated at 37°C (the same temperature used for RPTEC) and observed for morphological aberrations over 48 hrs.

### Detection of cytomegalovirus by an indirect immunofluorescence assay (IFA)

A standard cytospin procedure was used to deposit RPTEC from a 48 hr culture onto a glass slide. IFA was performed using a commercial kit with a primary antibody directed against a CMV immediate early protein, and a secondary antibody that was labeled with fluorescein isothiocyanate (LIGHT DIAGNOSTICS™ CMV IFA Kit, Millipore, Billerica, MA).

### Electron microscopy of virus-contaminated RPTEC

The BGM of a five day RPTEC culture was replaced with fresh ice-cold cacodylate-buffered 4% gluteraldehyde (pH 7.2). After 2 hrs at room temperature, the fixed cells were scraped free using a cell scraper, and pelleted by centrifugation at 8,000 x g for 10 minutes. The fixative was removed, and the cell pellet resuspended with cold fixative to a final volume of 500 μl, then stored overnight at 4°C. The fixed cells were post-fixed with osmium tetroxide, stained with uranyl acetate, embedded in Spurr’s embedding medium, then thin-sectioned. The thin sections were stained with uranyl acetate and lead citrate and transmission electron microscopy performed using a Hitachi H-600.

### Isolation of adventitious viruses from five-day old contaminated RPTEC cultures

Five days after being seeded, about 50% of the RPTEC had completely deteriorated, whereupon spent BGM media was added to 2 groups of subconfluent A549, BHK-21, CV-1, HEK-293, LLC-MK2, MDCK, MDCK-London, Mv1 Lu, NIH/3 T3, Vero E6, and WI-38 cells in complete growth media, and to 2 groups of LLC-MK2 and MDCK and Mv1 Lu cells in serum-free media containing L-1-tosylamide-2-phenylethyl chloromethyl ketone (TPCK)-treated trypsin. The TPCK-trypsin was at a final concentration of 2 μg/mL (MDCK and MDCK-London cells) or 0.2 μg/mL (LLC-MK2 and Mv1 Lu). For each group, 1 set was incubated at 37°C, the other at 34°C (incubation at 2 different temperatures is standard in our laboratory, as many of the respiratory viruses we work with preferentially replicate at temperatures lower than 37°C). TPCK-trypsin in serum-free media was used to facilitate the isolation of influenza and other viruses that require protease cleavage of some viral component for infectivity. After inoculation, the cells were re-fed every 3 days with 3% serum media or serum-free media with trypsin for long-term (up to 30 day) observations.

### Isolation of adventitious viruses from frozen RPTEC cell-lysates

At day 7 post-seed, only about 10% of the RPTEC remained attached to the flask, a majority of which were vacuolated and showed other signs of CPE. To facilitate the isolation of viruses other than CMV, the cells were scraped free and transferred along with the spent BGM into a sterile 50 mL polypropylene centrifuge tube, and frozen at −20°C for one week (this step reduces the number of viable CMV virions by a factor of many logs, since CMV loses viability when stored at −20°C) [38]; [J. Lednicky, unpublished]. Next, the frozen tube of scraped RPTEC was freeze-thawed three times, alternating between freezing at −20°C for 12 hrs and a 30 minute thaw at room temperature, as an additional measure to further reduce the number of viable CMV particles. After the third thaw, an aliquot was tested using the cells and methods of section 2.5 above, and the remainder frozen at −80°C for retrospective analyses.

### PCR and RT-PCR for the detection of viruses

Intracellular DNA was purified from a 48 hr RPTEC culture using a QIAamp DNA mini kit (Qiagen, Valencia, CA) and tested by PCR for CMV, HHV-1 and −2, and polyomaviruses SV40 and BKV. Total RNA was purified from a freeze-thawed seven-day old RPTEC culture supernatant using a QIAamp Viral RNA kit (QIAGEN). The primers and conditions that were used for PCR-based detection of viruses were based on published literature and will be provided upon request. Since syncytia were formed by the second virus (not CMV) that we were attempting to identify, PCR efforts were focused on human herpes, paramyxo (measles, mumps, metapneumovirus, parainfluenza viruses 1–5, respiratory syncytial virus), and coronaviruses.

RT-PCR for RNA virus screens was performed with Omniscript reverse transcriptase (Qiagen) followed by PCR with Hotshot TAQ (New England Biolabs, Ipswich, MA) 68°C. HCoV-NL63 was first detected using a pancoronavirus RT-PCR assay for the *viral polymerase* gene with primer pair Cor-FW and Cor-RV [42], followed by sequencing of the 251 bp amplicon. That was accomplished using Cor-RV for cDNA synthesis (with reverse transcription performed for 1 hr at 37°C), and PCR performed as: initial denaturation step: 94°C (1.5 min); 30 cycles of 94°C (20 sec), 48°C (30 sec), 68°C (30 sec); terminal extension step at 68°C (3.5 min); 4°C ∞. For confirmation, primer pairs N5-PCR1 and N3-PCR1 [42] and repSZ-1, and repSZ-3 [33] were used with PCR parameters similar to those for Cor-FW and Cor-RV, and the resulting amplicons sequenced. N5-PCR1 and N3-PCR1 amplify a 314 bp amplicon from the HCoV-NL63 n*ucleocapsid* region. N3-PCR1 was used to generate cDNA, and PCR performed at an annealing temperature of 46°C. Following cDNA synthesis primed with repSZ-RT [33], primer pair repSZ-1, and repSZ-3 amplify a 237 bp amplicon from the HCoV-NL63 ORF1b region at a PCR annealing temperature of 46°C.

### Electron microscopy of LLC-MK2 cells infected with HCoV-NL63 from RPTEC

LLC-MK2 cells that were RT-PCR positive for HCoV-NL63 were trypsinized to detach them from the growing surface of a T75 flask, pelleted, and the pellet resuspended in ice-cold 4% paramormaldehyde, 2% gluteraldehyde, in 0.1 M sodium cacodylate, pH 7.2. They were subsequently analyzed as described above.

### Sequencing of HCoV-NL63 genome

Targeted HCoV-NL63/RPTEC/2004 sequences were RT-PCR-amplified from purified RNA using a genome walking strategy. Briefly, overlapping primers described by H. Geng *et al.* (GenBank JX524171) and others [33,42] were used to obtain the viral sequence. AccuScript High Fidelity Reverse Transcriptase (Agilent Technologies, Inc., Santa Clara, CA) was used for first-strand cDNA synthesis in the presence of SUPERase-In RNase inhibitor (Ambion). PCR was performed using Phusion Polymerase (New England Biolabs) with denaturation steps performed at 98°C. The 3′ and 5′ ends of HCoV-NL63/RPTEC/2004 were determined from vRNA using a RACE (rapid amplification of cDNA ends) kit (RLM RACE, Ambion, Austin, TX) following the manufacturer’s instructions. Sequences were analyzed using an Applied Biosystem 3130 DNA analyzer by using BigDye Terminator (v. 3.1) chemistry and the same primers used for amplifications.

### Molecular dataset, sequence alignment, and phylogenetic analysis

The genomic sequence for isolate NL63/RPTEC/2004/1 was combined with other representative NL63 genomic sequences [34] available in GenBank (ncbi.nlm.nih.gov/genbank/index.html) to build the final dataset. Full genome alignments were performed using Mafft 5.8 [73] followed by minor manual adjustments in ClustalW [74]. The E-INS-I alignment strategy was used with the following parameters: scoring matrix (BLOSUM62), gap open penalty (1.53), and offset value (0). The aligned dataset was imported into jModelTest version 0.1.1 [75] and the Akaike information criterion (AIC) was used to select a best-fit model of evolution for phylogenetic analysis. Phylogenetic trees were constructed using MrBayes 3.1.2 [76]. The Markov chain was run for a maximum of 10 million generations, with a stopping rule implemented so that the analysis would halt when the average deviation of the split frequencies was < 0.01. Four independent analyses were conducted, each with 1 cold and 3 heated chains with the default heating parameter (temperature = 0.2). Every 1000 generations were sampled and the first 25% of MCMC samples discarded as burn-in.

### HCoV-NL63/Amsterdam-1

HCoV-NL63/Amsterdam-1 was obtained from the Biodefense and Emerging Infections Research Resources Repository (BEI Resources, Manassas, VA).

### Plaque assays

Plaque assays were performed following the procedures outlines in references 39 and 50.

### New batch of primary human kidney cells

Primary human kidney cells were obtained from Lonza, Inc. (Allendale, NJ). The cells chosen were: Renal Cortex Epithelial Cells (HRCE) (Cat #: CC-2554, Lot #: 1 F2266, cryopreserved 13 Oct 2010), Human Renal Epithelial Cells (HRE) (Cat #: CC-2556, Lot #: 5 F1314, cryopreserved 19 Oct 2005), and Renal Proximal Tubule Epithelial Cells (RPTEC) (Cat #: CC-2553, Lot #: 0000203150, cryopreserved 21 Dec 2001). The primary cells were grown in Clonetics renal epithelial basal medium (REBM, Lonza, Inc.) (Catalog No: CC-3191, Lot #: 0000345705) with Clonetics REBM SingleQuots supplements (fetal bovine serum, gentamycin sulfate, amphotericin B, insulin, recombinant human epidermal growth factor, transferrin, hydrocortisone, epinephrine, and triiodothyronine).

## Competing interests

The authors declare that they have no competing interests.

## Authors’ contributions

JAL conceived of the work, participated in all procedures, interpreted data; TBW performed phylogenetic analyses, interpreted data, and both JAL and TBW wrote the manuscript, EM, JCL, SBH, and MCL performed cell culture and virology work, and photographed cells; EM assisted with DNA and RNA extractions, and IFA. JCL helped format the manuscript. All authors read and approved the final manuscript.
